# Gynecological Insights into Lynch Syndrome—A Comprehensive Review of Cancer Screening and Prevention

**DOI:** 10.3390/medicina60122013

**Published:** 2024-12-06

**Authors:** Elena Chitoran, Roxana-Elena Bohiltea, Vlad Rotaru, Cristiana-Elena Durdu, Madalina-Nicoleta Mitroiu, Laurentiu Simion

**Affiliations:** 1 Medicine School, “Carol Davila” University of Medicine and Pharmacy, 050474 Bucharest, Romania; chitoran.elena@gmail.com (E.C.);; 2 General Surgery and Surgical Oncology Department I, Bucharest Institute of Oncology “Prof. Dr. Al. Trestioreanu”, 022328 Bucharest, Romania; 3 Obstetrics, Gynecology and Neonatology Department, “Filantropia” Clinical Hospital, 011132 Bucharest, Romania

**Keywords:** Lynch syndrome, mutations of DNA mismatch repair genes, MMR genes, high-risk patients, cancer screening, gynecological cancers, prevention

## Abstract

Lynch syndrome, one of the most common genetic syndromes predisposing to cancer, is associated with a series of malignant conditions, among which the most frequent is colorectal cancer, but gynecologic cancers (especially endometrial) are also quite common. Despite the significant progress made in understanding this condition over time, there are still aspects in managing this condition that have not demonstrated clear benefits. This article aims to summarize the recommendations of international societies and present the latest developments in managing Lynch syndrome, focusing on gynecologic cancer screening and possible prevention strategies. Advances in genetic testing procedures and discoveries related to the association between oncological pathology frequency and the affected pathogenic variant type will probably lead to personalized medicine focused on the individual patient in the coming years. Although various screening methods for gynecological cancers in patients with Lynch syndrome have been used over time, they have not shown significant survival benefits. This highlights the need for studying and implementing new screening and diagnostic methods, which have been under investigation in recent years and are mentioned in this article.

## 1. Introduction

With a prevalence in the general population ranging from 1 in 100 to 1 in 180, Lynch syndrome (LS), known in the past as hereditary nonpolyposis colorectal cancer (HNPCC), plays a significant role in the predisposition to develop genetically influenced cancers [1]. LS is distinguished by its predisposition to a variety of cancers, with the colorectum and endometrium being the most commonly affected sites. Additionally, LS involves other organ sites, such as the pancreas, stomach, small intestine, brain, ovary, hepatobiliary tract, urinary tract, prostate, and sebaceous skin tumors [2,3]. Being a condition with autosomal dominant inheritance, LS is caused by the occurrence of mutations in the DNA mismatch repair (MMR) genes. The most commonly affected genes, mutations in which have the greatest clinical impact, are represented by the MLH1, MSH2, MSH6, PMS2, and EPCAM genes [4]. The frequency of cancers associated with LS varies depending on the affected gene. For instance, in the case of MLH1, the cumulative incidence of endometrial cancer by the age of 70 is 34%, whereas for mutations in the MSH2, MSH6, and PMS2 genes, it is 51%, 49%, and 24%, respectively. As for ovarian cancer, the cumulative incidences are 11% for MLH1, 15% for MSH2, and 0% for both MSH6 and PMS2 mutation carriers [3]. The type of affected gene, as well as the type of mutation, are important factors that influence the age of onset for these types of cancers [5].

## 2. Methods

In this article, we conducted a comprehensive literature review by systematically searching major academic databases, including Scopus, PubMed, and Web of Science. These platforms were chosen due to their extensive and reputable coverage of biomedical and clinical research. The search utilized key terms such as “Lynch syndrome”, “gynecological cancers”, “ovarian cancer”, “endometrial cancer”, “screening”, and “prevention”, which are central to the topic of this review. We specifically focused on articles and clinical guidelines published in English or Romanian, as these languages were accessible to us and ensured a relevant and high-quality source base. Sources published in other languages were excluded to maintain consistency and clarity in the review.

To ensure the quality and relevance of the included studies, we excluded case reports and case series, as these often lack generalizability and comprehensive data. Instead, we focused on systematic reviews, meta-analyses, and original research articles that provided broader insights into the research area. Systematic reviews and meta-analyses are particularly valuable in synthesizing data from multiple studies, offering a higher level of evidence. By concentrating on these types of articles, we aimed to provide a well-rounded and evidence-based overview of the current state of knowledge regarding Lynch syndrome and its connection to gynecological cancers, with an emphasis on screening and prevention strategies. This approach also helps avoid bias from single-case reports and small sample studies, ensuring a more reliable and generalized perspective.

## 3. Lynch Syndrome Diagnosis

The clinical identification of LS was initially reliant on clinical criteria (Amsterdam I and II criteria) introduced in the 1990s [6,7]. Despite possessing good specificity, these criteria lacked sensitivity, necessitating their revision into the Bethesda Criteria (Table 1). The updated criteria aim to expand the scope and identify patients who should undergo screening for LS [8]. As per the European guidelines from the EHTG and ESCP, the Amsterdam criteria and/or revised Bethesda criteria are insufficient to guide tumor testing due to their low sensitivity in detecting patients with Lynch syndrome. According to these guidelines, LS screening should be conducted for all patients with colorectal cancer. As such, all colorectal tumors should be tested using immunohistochemistry or for microsatellite instability. Subsequently, testing for MLH1 hypermethylation should be carried out if necessary [9].

The primary approach to identifying individuals with Lynch Syndrome (LS) typically involves analyzing colorectal and endometrial cancer tumor samples for microsatellite instability (MSI) or deficiencies in mismatch repair (MMR) protein expression, followed by confirmatory germline testing for those with indicative tumor test results. While effective for cancer diagnosis, this strategy has limitations, as it excludes the possibility of proactive cancer prevention in unaffected individuals [10].

To address these gaps, predictive models like PREMM5 have been developed and validated. This model leverages easily obtainable clinical information—such as age, sex, and personal and family cancer history—to estimate the likelihood of mutations across all five LS-associated genes. As an accessible online tool, PREMM5 facilitates quick risk assessments and enables healthcare professionals, including oncologists and gynecologists, to identify candidates for germline testing more efficiently [11].

Other tools, such as MMRpredict and MMRpro, have demonstrated improved sensitivity and specificity in identifying MMR pathogenic variant carriers [11,12]. Nevertheless, their effectiveness heavily relies on accurately recorded family self-reported history, which can be time-consuming and challenging to manage within busy clinical settings. As a result, these tools may not always meet the required specificity and sensitivity as a primary screening method for identifying MMR pathogenic variant carriers. However, in cases where tumor material is unavailable, these prediction models can serve as valuable resources to guide germline testing [13].

These predictive models offer significant potential in low-resource settings where tumor testing is not feasible. They serve as a practical alternative, guiding the prioritization of germline testing for individuals at elevated risk of LS, ensuring a broader reach of preventive strategies in underserved populations. Despite some limitations in specificity and sensitivity, these models represent an invaluable resource for integrating genetic risk assessment into routine care in such environments [14,15].

The 2020 guidelines recommend genetic testing for all patients diagnosed with endometrial cancer (EC) [16]. Therefore, to identify the mismatch repair deficiency, immunohistochemistry should be used. In the event that immunohistochemistry testing shows a mutation in MSH6, MSH2, or PMS2, further confirmation of LS should be conducted through germline genetic testing. If the IHC testing indicates a mutation in MLH1 or both MLH1 and PMS2, the diagnostic algorithm involves testing for MLH1 hypermethylation. The absence of MLH1 hypermethylation necessitates germline genetic testing. For cases where IHC shows abnormality with the loss of MSH2, MSH6, or isolated PMS2 protein expression, germline genetic testing should be offered to confirm LS. In the case of colorectal cancer, both the screening and genetic testing of relatives have proven effective in reducing mortality among LS patients. Considering that colorectal cancer has a comparable incidence to endometrial cancer in LS patients, universal screening of endometrial tumors is justified and is supported by numerous international groups and societies, with the Manchester Consensus Group being one of them [17]. The International Society of Gynecological Pathology (ISGyP) recommends testing for MMR status/MSI in all endometrial carcinoma samples, regardless of age [18]. Another society that considers universal screening of endometrial tumors beneficial is the British Gynecological Cancer Society (BGCS) [19].

The ACOG guidelines do not provide strict recommendations regarding genetic testing of endometrial and colorectal tumors and offer multiple alternatives for clinicians. One initial option is universal screening. Another option is to conduct screening based on the analysis of patients’ personal and familial history. Lastly, the third option is represented by tumor testing for all endometrial or colorectal tumors diagnosed before the age of 60 years [20].

Regarding ovarian cancer (OC) in the context of LS, clinical management is guided by limited recommendations due to insufficient clinical data. A study conducted at a single center revealed that 21% of non-serous epithelial OC exhibited MMR deficiency through IHC [21]. There are several differences between sporadic ovarian cancer (OC) and OC in the context of LS. When we talk about OC within LS, it is typically diagnosed in younger patients and at a less advanced stage, leading to a better prognosis. The predominant histologic subtype in this case is usually endometrioid [5]. The data indicate that 7% of patients diagnosed simultaneously with both endometrial and ovarian cancer have LS [22]. Recent recommendations from several professional organizations advise testing all epithelial OC patients for BRCA1/2 pathogenic variants [23,24]. Testing premenopausal patients diagnosed with epithelial OC for both BRCA1/2 and Lynch syndrome is justified, as the cumulative risk of OC in these conditions is similar. Technological advancements also allow for testing multiple genes without significant additional financial burdens [17].

Other gynecological cancers, such as uterine leiomyosarcoma or squamous cell cancers of the cervix, vulva, or vagina, do not have a proven association with LS. The primary cause of these cancers is persistent infection with high-risk human papillomavirus (HPV) [25,26,27,28,29,30]. Therefore, these types of cancers are not typically within the category requiring testing for LS. An exception arises with HPV-negative endocervical adenocarcinoma, which presents challenges in the differential diagnosis from lower-segment endometrial tumors [31].

Even though a significant portion of breast cancer cases can be prevented through screening, it remains one of the leading causes of mortality among women [32]. Regarding the association between LS and breast cancer, a study involving 60,466 patients did not show a statistically significant association between mutations in the MLH1, MSH2, and EPCAM genes and breast cancer. However, a modest association was observed in the case of mutations in the MSH6 gene (*p* = 0.013) [33]. In another study involving 32,247 patients, the mismatch repair genes (MLH1, MSH2, and MSH6) were not found to be significantly associated with an increased risk of breast cancer in the overall analyses of the participants [34].

**Table 1:** Amsterdam II and Bethesda criteria [35].

Amsterdam II Criteria (The Presence of All the Criteria Is Required)	Bethesda Criteria (A Minimum of One Criterion Is Required)
A minimum of three family members affected by cancers within the HNPCC narrow spectrum.	Colorectal cancer diagnosis before the age of 50.
Among these three affected individuals, at least one should be a first-degree relative.	A second synchronous colorectal cancer or a metachronous colorectal cancer, irrespective of age.
The condition should involve at least two generations.	A second cancer from the wide range of HNPCC-associated cancers, regardless of age.
The presence of at least one cancer before the age of 50.	Pathological findings indicating microsatellite instability occurring before the age of 60.
There should be no history of familial adenomatosis coli.	Colorectal cancer with at least one first-degree relative having a cancer from the wide range of HNPCC-associated cancers before the age of 50.
	Colorectal cancer with at least two first-degree relatives having a cancer from the wide range of HNPCC-associated cancers, regardless of age.

## 4. Screening for Gynecological Cancers in Patients with Lynch Syndrome

Presently, there are no standardized surveillance guidelines for LS patients due to insufficient evidence demonstrating their clinical benefits in comparison to focusing on raising awareness and promptly investigating “red flag symptoms”, such as abnormal vaginal bleeding, pelvic and abdominal discomfort, weight loss, changes in bowel habits, and recurrent urinary symptoms [17]. More so, the quality of life in oncologic patients should be carefully monitored from the beginning of the diagnosis [36,37,38], and with new targeted therapies that contribute to a significant improvement in overall survival and progression-free survival appearing in recent years [39,40] and becoming routine [41], screening and early detection become paramount in patients with genetic predisposing conditions for malignant disease. Also, information on the correlations between various conditions may result in shaping a more informed and compassionate healthcare system [42] and highlighting significant clinical heterogeneity and the need for tailored management strategies [43]. Endometrial biopsy and transvaginal ultrasound (TVS) are the two most frequently employed methods for surveillance. TVS has demonstrated good sensitivity in detecting endometrial cancer in asymptomatic postmenopausal women [44]. As the thickness of the endometrium in premenopausal women is influenced by several factors, such as the use of oral contraceptive pills, menstruation, and intermenstrual bleeding [45], relying solely on TVS for LS surveillance is inadequate [46]. Numerous studies have indicated that endometrial biopsy is more effective in detecting (pre)malignant lesions of the endometrium compared to TVS alone [47,48]. Additionally, several reports have suggested that combining TVS with endometrial biopsy could enhance the effectiveness of surveillance [49,50,51].

The Manchester International Consensus Group made recommendations regarding the gynecological surveillance of patients with LS. Thus, patients should attend an annual medical visit starting at the age of 25, during which warning signs, as well as issues related to contraception and fertility, should be discussed. Moreover, invasive gynecological surveillance is not recommended because it has not demonstrated benefits [17]. The 2018 FIGO Cancer Report recommends using transvaginal ultrasound together with endometrial aspiration biopsy as a screening method for EC, starting at the age of 35, and performing these examinations annually until prophylactic hysterectomy [52]. The 2021 guidelines from the ESGO (European Society of Gynecological Oncology)/ESTRO (European Society for Radiotherapy and Oncology)/ESP (European Society of Pathology) recommend using annual transvaginal ultrasound along with annual or biennial endometrial biopsy as a screening method starting at the age of 35 [53]. The 2021 guidelines from the British Gynecological Cancer Society (BGCS) mention the possibility of using annual hysteroscopy in addition to transvaginal ultrasound and endometrial biopsy [19]. The guidelines of the Romanian Society of Obstetrics and Gynecology in 2022 recommend annual transvaginal ultrasound and endometrial biopsy as follows: starting at 30 years of age for patients with MSH2 mutations, starting at 35 years of age for patients with MLH1 mutations, and starting at 40 years of age for patients with MSH6 mutations. Women with heterozygous PMS2 mutations do not require gynecological surveillance as their absolute risk of gynecological cancer is very low [54]. Guidelines from international societies are listed in Table 2.

Additional alternative surveillance methods are currently being researched, such as vaginal tampon-based endometrial sampling, Pap test, and urinary biomarkers for detecting EC. Vaginal tampon-based endometrial sampling is a noninvasive approach that enables patients to perform home-based self-sampling, yet its suitability and efficacy require further investigation [59]. One of the advantages of this method is that, being less painful than traditional approaches, it is more widely accepted. Although adherence to screening among women at increased risk for Lynch syndrome has improved with the use of this method, further research is needed to establish its effectiveness and safety [59]. According to a systematic review, the Pap test was found to have low sensitivity as a diagnostic method. Nonetheless, advanced genetic and epigenetic technologies offer a promising outlook for improving its diagnostic accuracy [60]. Moreover, urinary biomarkers for detecting endometrial cancer, utilizing cytology, spectroscopy, transcriptomics, metabolomics, and proteomics, have demonstrated potential clinical advantages. Urine contains substances that can contribute to the diagnosis of endometrial cancer, including malignant cells, tumor DNA, secreted extracellular vesicles, proteins, and peptides. To explore these markers, techniques such as genomics, proteomics, spectroscopy, single-cell technology, cytology, and transcriptomics can be employed. This is possible due to the fact that all of these systemic biomarkers reach the kidneys and are subsequently released into the urine [61].

Among gynecological cancers, ovarian cancer remains the one with the highest mortality rate [62]. Despite extensive efforts, there has been minimal progress in reliably detecting ovarian cancer at an early, more curable stage. Over time, methods such as measuring serum markers, pelvic clinical examination, and ultrasound have been studied for OC screening both in the general population and within high-risk categories. However, no method has proven effective in increasing survival rates. As a result, there is no recommendation for routine OC screening from any national organization [63]. CA125 measurements and/or transvaginal ultrasound (TVU) have been the most commonly tested methods, but their success has been limited thus far [64]. CA125 tumor antigen has played a crucial role in managing OC patients, primarily by assessing the response to therapy and monitoring for recurrent disease after treatment. However, CA125 is not a reliable screening marker due to its potential for both false-positive and false-negative results. Several factors, including race/ethnicity, age, hysterectomy, smoking history, and obesity, can influence CA125 levels [63]. Additionally, some data indicate that even the combined use of CA125 and transvaginal ultrasound has not led to increased survival in the general population [12].

As per the 2014 ACOG guidelines, there is no established guideline for monitoring patients with OC in the context of LS. Moreover, the results of OC surveillance in women with BRCA1 and BRCA2 mutations may not be directly applicable to Lynch syndrome due to significant biological differences between OC in Lynch syndrome and OC seen in hereditary ovarian and breast cancer syndrome [20]. More society guidelines on OC screening in patients with LS are listed in Table 3.

Even though there are currently no firm recommendations or highly effective methods for ovarian cancer screening in patients with Lynch syndrome, innovative diagnostic approaches are being developed and show promise in improving outcomes. One promising technique is the PapSEEK test, which utilizes PCR to analyze DNA extracted from routine Pap smears. This approach targets 18 specific genetic mutations or chromosomal abnormalities and demonstrates a 33% sensitivity in detecting ovarian cancer. The sensitivity improves to 45% when intrauterine sampling is performed using a Tao brush. Additionally, applying the same methodology to circulating plasma DNA results in a 43% sensitivity. When both techniques are combined, the detection rate increases significantly, reaching 63% [65].

Extensive research has explored the use of biomarkers for detecting ovarian cancer at earlier stages. Human Epididymis Protein 4 (HE4) stands out due to its elevated levels in about 70% of ovarian cancer cases and its greater specificity compared to CA125, as it is rarely elevated in benign ovarian tumors [66].

While CA125 is elevated in over 90% of advanced-stage ovarian cancers and 50–60% of early-stage cases, the combination of HE4 and CA125 enhances diagnostic accuracy, achieving 76.4% sensitivity and 95% specificity [67]. Other biomarkers, such as CA72.4 and CA15.3, have been studied alongside CA125 but have shown no significant improvement in early detection compared to CA125 alone [68,69,70,71].

Simultaneously, advancements in imaging techniques, such as magnetic resonance imaging (MRI), magnetic relaxometry (MRX), and microbubble-enhanced transvaginal ultrasound, are improving the ability to detect small ovarian tumors [72,73]. These methods, alongside novel technologies like light-induced autofluorescence and TP53 autoantibody analysis, represent a significant step forward in addressing the challenges of early ovarian cancer detection. However, further research is essential to integrate these promising tools into routine clinical practice [74].

## 5. Gynecological Cancer Prevention in Patients with Lynch Syndrome

Regarding the prevention of gynecological cancers in patients with LS, this can be achieved through surgical means or chemoprevention (Table 4). In the context of LS, at the age of 40, the risk of EC is 2–4%, while the risk of OC is 1–2%. At the age of 50, these risks increase to 8–17% for EC and 3–7% for OC [75]. According to ACOG guidelines, surgical prophylaxis can be carried out through hysterectomy and bilateral salpingo-oophorectomy, which should be considered for patients aged 40–45 who have completed their family planning [20,76]. This recommendation is also supported by ESGO/ESTRO/ESP guidelines. The timing and options of risk-reducing surgery (RRS) can be personalized based on individual factors such as childbearing potential, existing health conditions, menopause status, family history, and the specific germline pathogenic variants. For carriers of pathogenic MSH6, it is recommended to undergo RRS after reaching 40 years of age. Minimally invasive options should be offered to the patients at any stage of the disease [77]. On the other hand, carriers of either pathogenic MLH1 or pathogenic MSH2 may consider risk-reducing surgery around the age of 35 years when fertility is no longer a concern. However, it is essential to note that the role of RRS for pathogenic PMS2 carriers requires further evaluation due to their relatively lower risk of endometrial cancer [78]. According to the guidelines set forth by the British Gynecological Cancer Society, the timing of risk-reducing surgery for carriers of Lynch syndrome pathogenic variants is tailored to the specific gene involved, based on the associated cancer risks and typical age of onset. Surgery is advised starting at age 35 for individuals carrying mutations in the MLH1 or MSH2 genes due to their earlier onset of cancer risk. For carriers of MSH6 mutations, surgery is recommended from age 40, reflecting the slightly later onset of significant risk. Finally, for PMS2 mutation carriers, who generally face the lowest and latest risk, surgery is deferred until after the age of 50. Data from the Prospective Lynch Syndrome Database indicate that individuals carrying heterozygous pathogenic PMS2 variants do not exhibit an elevated risk of developing colorectal, endometrial, or ovarian cancer prior to the age of 50. Beyond this age, the associated risks appear to increase only marginally, highlighting a relatively lower risk profile compared to other Lynch syndrome-associated gene mutations [79].

Both the traditional surgical approach and minimally invasive techniques can be used for surgical prophylaxis. The laparoscopic approach is favored in uncomplicated cases, where resources permit, due to its association with reduced postoperative pain, faster recovery, and improved short-term quality of life [81,82]. Prior to a hysterectomy, it is important to ensure that colonoscopy screening is up-to-date. Preoperative endometrial biopsy is justified as occult neoplastic lesions have been identified during prophylactic surgery. Intraoperative examination of the excised specimen can also be useful. Identifying occult neoplasms in the uterine tubes necessitates complete resection of the tubes in patients opting for hysterectomy with ovarian preservation [83].

Risk-reducing oophorectomy in premenopausal women leads to surgical menopause and is associated with various symptoms and health risks. These include vasomotor symptoms, reduced sexual function, urogenital dryness, cognitive decline, emotional lability, and increased risks of osteoporosis, cardiovascular disease, and CRC [84]. In this situation, hormone replacement therapy is necessary until around the age of 51 to counteract these effects [17].

According to the ACOG, the use of progestin-based contraception, including oral contraceptives, may also be considered as a chemopreventive strategy for EC in context of LS [20]. According to the British Gynaecological Cancer Society guidelines, aspirin has been shown to reduce cancer risk in Lynch syndrome, and its use has demonstrated good safety data in young adults. However, the optimal dose for cancer chemoprevention is still uncertain and requires further investigation. Alternative risk-reducing strategies, such as the LNG-IUS and other progestin preparations, are also being studied. These strategies are expected to reduce endometrial cancer risk in Lynch syndrome by promoting endometrial decidualization, atrophy, and apoptosis. Currently, there is insufficient data to support the effectiveness of these methods. Further research is needed in this area [19].

At present, there is no consensus regarding chemoprevention for colorectal cancer. Recommendations from various medical societies, including the Cancer Council of Australia (CCA), the National Comprehensive Cancer Network (NCCN), and the National Institute for Health and Care Excellence (NICE), advocate for the prophylactic use of aspirin to reduce colorectal cancer risk. European guidelines issued by the European Hereditary Tumour Group (EHTG) and the European Society of Coloproctology (ESCP) for Lynch syndrome provide additional details regarding the recommended aspirin dosage, specifying a daily dose of at least 75–100 mg, with necessary adjustments for overweight and obese individuals. Regarding the age at which aspirin administration should begin, the CCA Clinical Guidelines offer further clarification, recommending the initiation of aspirin treatment concurrent with the first colonoscopy, which typically occurs around the age of 25 years [85].

Regarding modifiable lifestyle factors, the World Cancer Research Fund (WCRF) guidelines for cancer prevention highlight several recommendations. These include maintaining an optimal body weight, with a BMI between 18.5 and 24.9, and sustaining a physically active lifestyle, ideally engaging in moderate-intensity physical activity. A balanced diet is also emphasized, comprising fruits, vegetables, and whole grains, while limiting the intake of fast food, red meat, sugar, and alcohol. Additionally, there is a strong recommendation for new mothers to breastfeed their newborns [86].

A meta-analysis revealed that aspirin has the potential to reduce the mortality related to several common cancers, including colorectal, prostate, lung, esophageal, pancreatic, brain, and stomach cancer [87]. The daily administration of 600 mg of aspirin could potentially be an effective method for preventing colorectal cancer in patients with Lynch syndrome, as indicated by the results of the CAPP2 study. Additionally, the study observed a decrease in the incidence of endometrial cancer (EC) in females who received aspirin compared to those in the placebo group. However, no significant difference was observed in non-CRC LS-associated tumors between the aspirin and placebo groups [88]. While the evidence is not as extensive as for gastrointestinal cancers, there is a growing indication of potential benefits from aspirin in ovarian malignancies. Research data point towards a potential inhibitory effect of aspirin on the development of EC, especially in obese individuals. However, at present, there is not enough data to recommend the use of aspirin as an adjuvant treatment for gynecological cancers outside of a clinical trial [89].

Certain risk factors such as smoking, high BMI, and alcohol consumption could potentially increase the risk of colorectal cancer in patients with Lynch syndrome. On the other hand, the effects of these factors are unknown regarding the risk of developing endometrial and ovarian cancers [90]. Another interesting and yet theoretical approach to prevention involves exploiting the innate immunosurveillance phenomenon observed in LS patients. Even cancer-free LS patients have been found to possess circulating cytotoxic T-cells that target MSI-induced frameshift neoantigens [91,92]. This type of biology has sparked speculation that immune-based therapies, such as immune checkpoint inhibitors and vaccination, could serve as primary cancer prevention measures for LS individuals, potentially offering protection against various cancer types. Clinical trials, like NCT03631641, have begun to explore the effectiveness and safety of these innovative approaches [93,94].

A particular aspect in Lynch syndrome patients is represented by the importance of Preimplantation Genetic Testing for Monogenic/Single-gene Disorders (PGT-M), a procedure utilized in assisted reproductive technology to prevent the transmission of genetic diseases to offspring. People of reproductive age, especially those considering parenthood, should undergo a comprehensive consultation involving experts in genetics and reproductive medicine. Interestingly, PGT-M is less commonly used for genes associated with Lynch syndrome compared to the breast cancer susceptibility genes BRCA1/2, even though both conditions have similar prevalence and severity [95].

## 6. Conclusions

Despite significant advancements in understanding cancer risk and the potential to reduce it in individuals with Lynch syndrome, there are still many aspects to explore in order to decrease the associated morbidity and mortality. Better detection of high-risk groups for Lynch syndrome is necessary and can be achieved through technological advancements, improved patient access to medical services, reimbursement for services like universal tumor screening, and the continuous enhancement of screening methods. The same applies to screening for Lynch-related cancers in diagnosed patients, as the benefits of screening for endometrial and ovarian cancer mortality are still being evaluated. Following a diagnosis of LS, patients should be included in the national oncogenetics registry, along with their first- and second-degree relatives, who should be encouraged to undergo genetic testing. Screening centers should have adequate resources, including geneticists and necessary equipment for immunohistochemistry (IHC) and polymerase chain reaction (PCR). It is essential for the oncogenetics network to closely collaborate with the national screening network. The study of new screening methods, the exploration of novel therapeutic approaches such as vaccine development, and the possibility of preventing the transmission of the condition to offspring through preimplantation genetic testing are just a few aspects that should be considered by clinicians to improve the prognosis of these patients.

## Figures and Tables

**Table 2:** Society guidelines on endometrial cancer screening in Lynch syndrome.

Society	Method
ACOG (The American College of Obstreticions and Gynecologists) 2014 [20]	Endometrial biopsy every 1–3 years, starting at 30–35 years of age
FIGO (International Federation of Gynecology and Obstetrics) 2018 [52]	Transvaginal ultrasound and endometrial aspiration biopsy starting at the age of 35 annually
ESGO (European Society of Gynaecological Oncology)/ESTRO (European Society for Radiotherapy and Oncology)/ESP (European Society of Pathology) 2021 [53]	Annual transvaginal ultrasound and annual or biennial endometrial biopsy starting at the age of 35
British Gynaecological Cancer Society (BGCS) 2021 [19]	Annual transvaginal ultrasound, hysteroscopy, and/or endometrial biopsy starting at the age of 35
Romanian Society of Obstetrics and Gynecology (SOGR) 2022 [39]	Annual transvaginal ultrasound and annual or biennial endometrial biopsy until the decision for hysterectomy is made (MSH2 starting from an age of 30 years, MLH1 starting from an age of 35 years, and MSH6 starting from an age of 40 years) In the case of heterozygous PMS2 mutations, the risk of gynecological cancers is very low. Therefore, these patients do not require screening.
Delphi Initiative for Early-Onset Colorectal Cancer (DIRECt) [55]	Pelvic examination and endometrial biopsy annually, beginning at the age of 30 to 35 years
The Manchester International Consensus Group [17]	Annual medical visit starting at the age of 25 Invasive gynecological surveillance is not recommended
American Society of Colon and Rectal Surgeons 2017 [56]	Pelvic examination and endometrial sampling annually, starting at the age of 30–35
American College of Gastroenterology 2015 [10]	Transvaginal ultrasound and endometrial biopsy starting at the age of 30–35
US Multi-Society Task Force on Colorectal Cancer 2014 [57]	Pelvic examination and endometrial sampling annually starting at the age of 30–35
European Society for Medical Oncology 2019 [58]	Annual gynecological examination, TVU, and endometrial biopsy starting at the age of 30–35

**Table 3:** Society guidelines on ovarian cancer screening in patients with Lynch syndrome.

Society	Method
ACOG (The American College of Obstreticions and Gynecologists) 2014 [20]	There is no established guideline for monitoring patients with OC in the context of LS The effectiveness of transvaginal ultrasound or CA125 for screening in these patients has not been proven.
American Society of Colon and Rectal Surgeons [56]	Transvaginal ultrasound annually starting at age 30–35
American College of Gastroenterology 2015 [10]	Transvaginal ultrasound annually starting at age 30–35
US Multi-Society Task Force on Colorectal Cancer 2014 [57]	Transvaginal ultrasound annually starting at age 30−35
European Society for Medical Oncology 2019 [58]	Annual gynecological examination, TVU with CA 125 analysis from age 30 to 35 years

**Table 4:** Society guidelines on the prevention of gynecological cancers in patients with Lynch syndrome.

Society	Recommendation
ACOG (The American College of Obstreticions and Gynecologists) 2014 [20]	Prophylactic hysterectomy and bilateral salpingo-oophorectomy in women who have completed their family planning or at the age of 40–45 years Progestin-based contraception, including oral contraceptives
British Gynaecological Cancer Society (BGCS) 2021 [19]	Risk-reducing surgery is recommended for MLH1 and MSH2 pathogenic variant carriers starting at the age of 35 years, for MSH6 pathogenic variant carriers from the age of 40 years, and for PMS2 pathogenic variant carriers after the age of 50 years Aspirin Levonorgestrel-releasing intrauterine system (LNG-IUS) and other progestin preparations
American Society of Colon and Rectal Surgeons 2017 [56]	Prophylactic hysterectomy and bilateral salpingo-oophorectomy in women who have completed their family planning or at age 40 years Aspirin
American Gastroenterological Association 2015 [80]	Aspirin
American College of Gastroenterology 2015 [10]	Prophylactic hysterectomy and bilateral salpingo-oophorectomy in women who have completed childbearing, optimally at age 40–45 years Aspirin
US Multi-Society Task Force on Colorectal Cancer 2014 [57]	Hysterectomy and bilateral salpingo-oophorectomy should be recommended to women who have finished childbearing or at age 40 years Aspirin
European Society for Medical Oncology 2019 [58]	Prophylactic hysterectomy with bilateral oophorectomy for women who have completed childbearing or are postmenopausal Aspirin

## Data Availability

No new data were created. All data supporting this article are available online.
